# Comparison of In Vitro and In Silico Assessments of Human Galactose-1-Phosphate Uridylyltransferase Coding Variants

**DOI:** 10.7759/cureus.33592

**Published:** 2023-01-10

**Authors:** Jimmy T Mitchell, Eric B Johnson

**Affiliations:** 1 Department of Clinical Sciences, Alabama College of Osteopathic Medicine, Dothan, USA; 2 Department of Anatomy and Molecular Medicine, Alabama College of Osteopathic Medicine, Dothan, USA

**Keywords:** galactosemia, molecular dynamic simulation, variant of uncertain significance, galactose-1-phosphate uridylyltransferase, galt

## Abstract

Introduction

Human pathogenic coding variations of the galactose-1-phosphate uridylyltransferase (GALT) gene cause classic galactosemia, a recessive disease of galactose metabolism. Unfortunately, there are many variants of uncertain significance (VUS) that need to be characterized in order to be able to predict the likelihood of classic galactosemia for all possible genotypes. There are many bioinformatic resources available that attempt to predict the pathogenicity of a human variant, but it is unclear if these methods realistically predict the consequence of these variants. To determine the clinical application of these resources, we compared the results of *in vitro* enzymatic assays with *in silico* predictive models.

Methods

In all assays, we compared the activity of the three human GALT VUS (Alanine81Threonine, Histidine47Aspartate, Glutamate58Lysine) to native GALT (nGALT) and to a variant of known pathogenic clinical significance (Glutamine188Arginine). The enzymatic activities of VUS recombinant proteins were compared to the results of *in silico *analytical methods. The* in silico* methods included the comparison of molecular dynamic simulation root-mean-square deviation (RMSD) results and the results from predictive programs PredictSNP, evolutionary model of variant effect (EVE), ConSurf, and sorting intolerant from tolerant (SIFT).

Results

The enzymatic assays showed that the variants tested had diminished Vmax relative to the native protein. The VUS RMSD data for both the whole protein and individual residues in the molecular dynamics simulations were not significantly different when compared to nGALT. The other predictive programs had mixed results for each VUS and were not consistent with the enzyme activity or simulation results.

Conclusions

Our experiments indicated a statistically significant decrease in enzymatic activity of the VUS when compared to nGALT. These experiments also demonstrated significant differences between* in silico* predictions and* in vitro* results. These results suggest that the in silico tools used may not be beneficial in determining the pathogenicity of GALT VUS.

## Introduction

Galactose-1-phosphate uridylyltransferase (GALT) is an enzyme responsible for converting ingested galactose to metabolically useful glucose. Attenuation or lack of GALT activity leads to the disease classic galactosemia. Classic galactosemia is inherited in an autosomal recessive fashion and affects one in 40,000-60,000 live births [1]. Patients affected by this disorder typically present with symptoms within the first few days of life once breastfeeding or formula feeding with dairy-based formula has begun. The accumulation of galactose and galactitol leads to the presenting symptoms, including failure to thrive, hepatic damage and dysfunction, cataracts, sepsis, and neurologic developmental delay. Diagnosis consists of measuring enzyme activity within red blood cells followed by genetic testing. There is currently no known cure or completely effective treatment. Treatment primarily targets symptoms and mainly consists of dietary restrictions. Even with early diagnosis and treatment, patients usually suffer from lifelong complications of progressive neuropsychiatric impairments [2].

There are over 300 GALT mutations that are confirmed to cause classic galactosemia, but dozens of other variants have been documented with unknown clinical significance [3]. A rising trend in medical research has been the use of computer-generated predictive models to detect and categorize the impact of mutations on protein structure and enzymatic activity. Utilizing predictive modeling, researchers can potentially predict which mutations are most likely to cause enzymatic dysfunction and lead to disease [4]. This information can help guide research strategies to be more efficient and resource-preserving by focusing on the most impactful mutations.

Several *in silico *methods have been established to analyze the structural consequence of changing an amino acid residue in a protein normally caused by a single-nucleotide polymorphism (SNP) [5]. PredictSNP is a consensus classifier that uses several variant prediction programs described below to predict the pathogenicity of protein-coding variations [6]. PredictSNP is useful because it incorporates the results from all the tools into one pathogenicity prediction. The results from a PredictSNP analysis also include the results from Multivariate Analysis of Protein Polymorphism (MAPP), which scores variants based on evolutionary and physiochemical analysis [7]; Predictor of human deleterious single nucleotide polymorphisms (PhD-SNP), which combines evolutionary and support vector machine methods to score variants [8]; and Polymorphism Phenotyping v2 (PolyPhen-2), which predicts the possible impact of an amino acid substitution using sequence and structural predictive calculations [9,10]. The evolutionary model of Variant Effect (EVE) predicts pathogenicity based on deep machine learning trained on sequences from thousands of species [11]. ConSurf is a bioinformatics tool for estimating the evolutionary conservation at each residue site and rates the impact of residue substitution [12-15]. Sorting Intolerant From Tolerant (SIFT) predicts the effects on the protein function of an amino acid substitution based on tangible properties of amino acids and sequence homology. SIFT can be utilized with naturally occurring and laboratory-induced missense mutations [16].

There have been various methods used to analyze GALT variants. Most often variants are found in genetic association screens and the pathogenicity is determined by statistical methods [17]. Some of the genetic screen results are complimented by red blood cell enzyme activity [18]. Other researchers have focused on computer simulation and predictive modeling [19]. Only a few studies have compared *in silico* analysis to enzyme kinetic analysis [20].

To better understand the effects of various mutations on the GALT enzyme, we conducted* in vitro* enzymatic studies and compared these results to numerous *in silico *bioinformatic models. We designed our research to bridge the gap between* in vitro* and *in silico* experiments. Utilizing data from enzymatic assays as well as numerous predictive modeling programs, we investigated the impact of variants of the unknown clinical significance of GALT. Understanding how these mutations impact the function of GALT could help in furthering understanding, guide the development of improved treatments, and possibly point toward a cure.

## Materials and methods

In vitro

MilliporeSigma Novagen HMS174 (DE3) Competent *Escherichia* *coli *cells were transformed with expression plasmids, pET-28a(+), produced by Twist Biosciences Inc., that express GALT variants with an amino-terminal 6xHis tag [21]. GALT protein induction and purification were performed using the QIAexpress® Ni-NTA Fast Start kit and ThermoFisher Scientific™ Halt™ Protease Inhibitor Cocktails using a procedure based on QIAexpress® Ni-NTA Fast Start Handbook using a lac operon expression system and nickel columns [21]. To verify a successful transformation, induction, and purification process, we utilized SDS-PAGE protein electrophoresis followed by Coomassie staining to confirm the presence of GALT protein in our samples. Using an already established assay [20], we performed a double displacement assay and detected the reaction rate using a BioTek plate reader to determine the activity of GALT over a 30-minute time period. The results were compared using ordinary one-way ANOVA.

In silico

Data from molecular dynamics simulation, PredictSNP, EVE, SIFT, and ConSurf were used to determine if a GALT variant may contribute to galactosemia. For molecular dynamics simulation, the human GALT structure was produced through homology modeling using the YASARAstructure program. The appropriate residues were changed to create the structures of the human variants analyzed and then the structures were run through the em_runclean macro. Three replicates were produced for these structure files by sequential runs of energy minimization. The GALT molecular dynamics simulations were run with YASARAstructure [22]. Optimization of the hydrogen bonding structure was used to increase the stability of the solute. A pKa prediction was also used to calibrate the protonation states of protein residues at a pH of 7.4 [23]. NaCl ions were added with a physiological concentration of 0.9%, with an excess of either Na or Cl to neutralize the cell. After the steepest descent and simulated annealing minimizations to remove clashes, the simulation was run for 20 ns using the YASARA force field for the solute and TIP3P for water [24]. The cutoff was 8 A for Van der Waals forces (the default used by AMBER, no cutoff was applied to electrostatic forces using the Particle Mesh Ewald algorithm) [25,26]. The individual residue RMSD data from the simulations were analyzed using two-way repeated measures ANOVA. The mean total protein RMSD for the last 5 ns was compared using ordinary one-way ANOVA.

Predictive modeling programs PredictSNP, EVE, Consurf, and SIFT were utilized to determine the pathogenicity of the variants examined. PredictSNP analysis was performed by submitting the P07902.fasta file derived from the Uniprot site https://www.uniprot.org/uniprotkb/P07902/entry to https://loschmidt.chemi.muni.cz/predictsnp1/ and the indicated variants were selected for analysis. The EVE analysis was performed by submitting the “GALT (GALT_HUMAN, P07902)” gene symbol for analysis on the evemodel.org website. ConSurf Data Base analysis was performed by submitting the 5in3 PDB ID on the https://consurfdb.tau.ac.il/ website. SIFT analysis was performed by submitting the P07902.fasta file to the https://sift.bii.a-star.edu.sg/www/SIFT_seq_submit2.html website and the indicated variants were selected for analysis.

## Results

In vitro analysis

There are many bioinformatic resources available that attempt to predict the pathogenicity of a human variant by using different analysis methods such as measuring protein stability, measuring residue conservation, machine learning, etc. [27]. It is unclear if any of these methods realistically predict the consequence of changing a residue in a protein and are thus useful in a clinical setting. To test the hypothesis that protein function matches the predicted pathogenicity of a variation using these resources, we compared the results of *in vitro* protein activity results to *in silico* predictive results.

Variants to test were chosen from ClinVar [28]. We chose the Alanine81Threonine (A81T), Histidine47Aspartate (H47D), Glutamate58Lysine (E58K), and variants of uncertain significance (VUS) because they were located in regions of the protein that had a small number of pathogenic or likely pathogenic variants. To validate that we could detect a pathogenic variant and to ensure that no residual bacterial GALT activity remained in our preparations, we chose to test the Glutamine188Arginine (Q188R) variant as a negative control.

**Figure 1:**
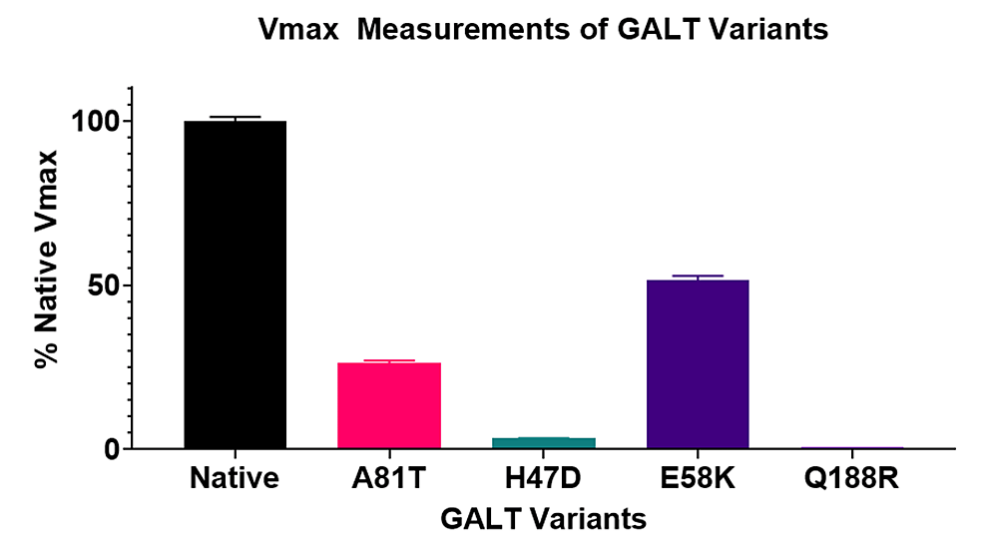
GALT Variant Activity. The production of glucose-1-phosphate was measured for each GALT variant through a series of enzymatic steps which result in the reduction of NADP to NADPH+ which is measured spectroscopically. The Vmax for each variant was compared to the Vmax of the native GALT protein using one-way ANOVA (p<0.01): GALT: galactose-1-phosphate uridylyltransferase; ANOVA: analysis of variance; ADP: nicotinamide adenine dinucleotide phosphate; NADPH+: nicotinamide adenine dinucleotide phosphate

When compared to native GALT (nGALT), all three experimental variants of uncertain significance tested had significantly reduced Vmax as analyzed with one-way ANOVA (p<0.01) (Figure 1). The variant A81T retained the most activity, with approximately half that of nGALT, 51.66%. The H47D variant exhibited 26.36% activity versus nGALT. The variant E58K had minimal enzymatic activity when compared to nGALT, 3.38%. The absence of activity seen with the Q188R negative control variant shows that there was little, if any, residual bacterial GALT contamination in the isolated protein preparations.

In silico analysis

**Figure 2:**
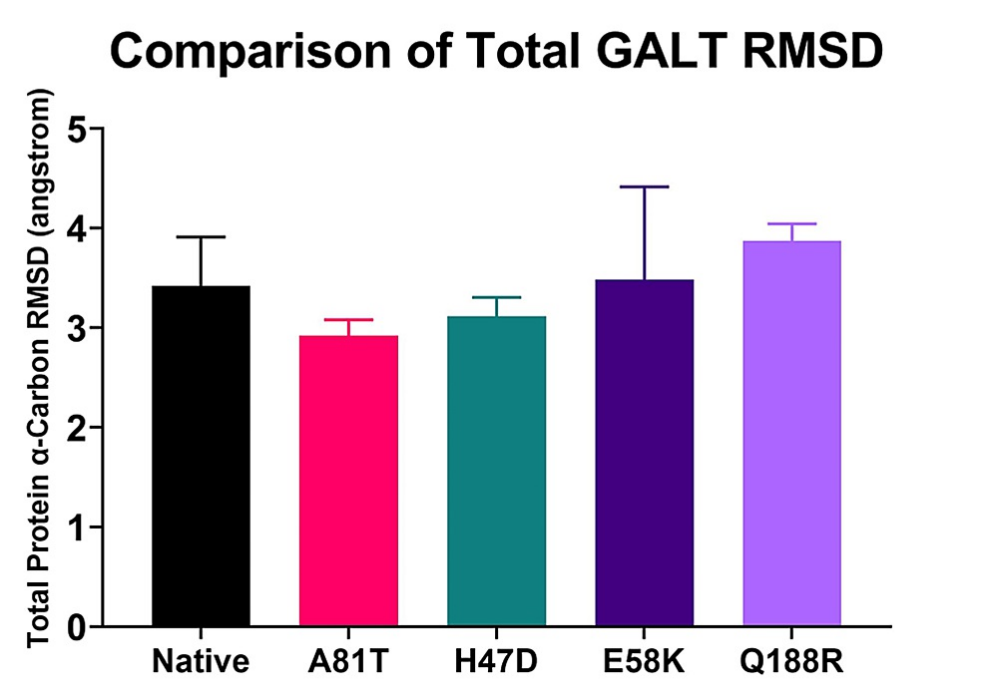
Total GALT RMSD Analysis. The PDB files representing each GALT variant were used in a 20-nanosecond simulation. The average RMSD value for the last five nanoseconds of the simulation were calculated and compared using an ANOVA test. All values were not statistically significant compared to native protein (p>0.01) GALT: galactose-1-phosphate uridylyltransferase; RMSD: root-mean-square deviation; PDB: Protein Data Bank; ANOVA: analysis of variance

Molecular dynamics simulation (MDS) was used to analyze variants of the GALT protein over a 20-ns time frame. To statistically compare the results, the last 5 seconds of the simulation were used to compare the variants. To test the total change in GALT shape during the simulation, the total RMSD of the alpha carbons of the protein were compared (Figure 2). In this analysis, the average RMSD value from 15 to 20 nanoseconds for each replicate was compared to the native GALT RMSD. An alternative analysis compared the RMSD values at each time point. Both analyses did not show any statistical significance using ANOVA analysis (p>0.01).

**Figure 3:**
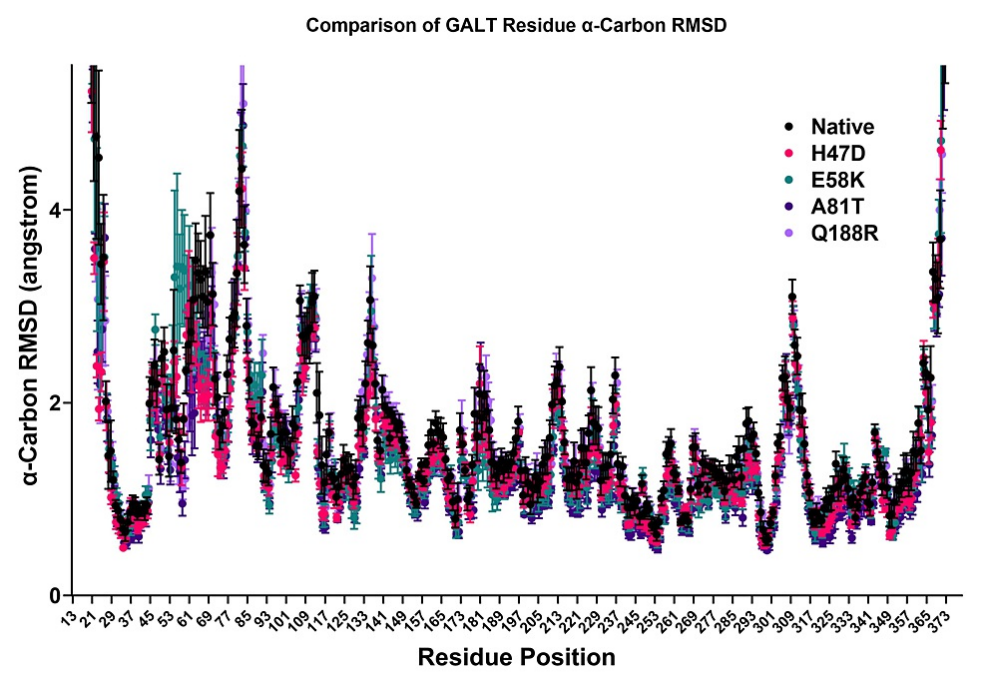
Residue RMSD Comparison. The PDB files representing each GALT variant were used in a 20-nanosecond simulation. The RMSD values for residues 13-373 during the simulation were calculated and compared using an ANOVA test. All values were not statistically significant (p>0.01). GALT: galactose-1-phosphate uridylyltransferase; RMSD: root-mean-square deviation; PDB: Protein Data Bank; ANOVA: analysis of variance

The change in RMSD of specific residues within a simulation could provide more mechanistically relevant information on the changes that occur with the introduction of a variation. Figure 3 shows that the pattern of residue RMSD is approximately the same as the variants and the native protein. There was no statistical difference found at any residue position.

Table 1 demonstrates the results from different bioinformatic programs that analyze protein variations. PredictSNP predicted that the A81T variation was neutral while the other variants were deleterious. The newly developed EVE program predicted that the A81T variation was benign and the Q188R variation was pathogenic but was unable to determine pathogenicity for H47D (uncertain) and E58K (no value assigned). Consurf shows that the experimental variants (A81T, E58K, H47D) all had flexibility in terms of residue choice at that position. The variant residues were within the list of residue varieties at those positions. In contrast, the control variant, Q188R, only tolerated a few residues at the 188 position and arginine was not one of them. SIFT predicted that all experimental variations would be tolerated while the Q188R variation would affect protein function.

**Table 1:** Comparison of Predictive Programs. Analysis of the H47D, E58K, A81T, and Q188R GALT variants using PredictSNP, EVE, Consurf, and SIFT. Pathogenic values are indicated by (-) and benign values are indicated by (+). Amino acid abbreviations: A=alanine, R=arginine, N=asparagine, D=aspartate, C=cysteine, E=glutamate, Q=glutamine, G=glycine, H=histidine, I=isoleucine, L=leucine, K= lysine, M=methionine, F=phenylalanine, P=proline, S=serine, T=threonine, W=tryptophan, Y=tyrosine, V=valine. EVE: Evolutionary model of Variant Effect; SIFT: Sorting Intolerant From Tolerant; GALT: Galactose-1-phosphate uridylyltransferase

	PredictSNP		EVE		ConSurf		SIFT	
Variant	Prediction	Expected Accuracy	Score	Classification	SCORE	Residue Variety	Prediction	Score
H47D	Deleterious (-)	0.87	0.56	Uncertain	-0.55	N,I,G,R,S,K,H,L,D,Q,E,T,A,Y	Tolerated (+)	0.21
E58K	Deleterious (-)	0.87	No value	No value	-0.28	L,R,S,H,Q,D,P,T,E,A,G,V,M,N,K,C	Tolerated (+)	0.08
A81T	Neutral (+)	0.75	0.26	Benign (+)	-0.30	P,L,E,T,R,S,F,N,I,C,K,D,Q,H,A,Y,W,M,G,V	Tolerated (+)	0.28
Q188R	Deleterious (-)	0.87	0.89	Pathogenic (-)	-1.388 (-)	H,F,Q	Affect protein function (-)	0.00

## Discussion

The purpose of this research was to compare variant pathogenicity predictive programs to empirical data from biochemical tests. We hoped to find a bioinformatic resource that would match the data seen in the biochemical assays. Matching or similar data would impart greater confidence in the bioinformatics resource, particularly in advising healthcare professionals of the patient’s likelihood of having a pathogenic genotype. In contrast, all the variants tested had decreased activity whereas the results from the bioinformatic resources were inconsistent or inconclusive.

The results from the biochemical assays of the variants were not expected. The original hypothesis was that the variants of uncertain significance in parts of the protein that does not have many pathogenic variants would likely be benign. In contrast, all the variants are potentially pathogenic. The A81T variant seemed to be the most functional variant with only a 48% decrease in activity. Correspondingly, PredictSNP and EVE both predicted this variation to be benign. It is unclear whether a 48% decrease in activity would be considered pathogenic. Perhaps this decrease in activity is tolerated and thus would not be present clinically. When considering other proteins involved in other diseases, the threshold at which a protein can decrease its activity and yet still be benign would likely be specific to the protein in question [29].

Many studies have found discrepancies with the predictive modeling of protein mutations [30]. Even with machine learning being a driving force behind newer modeling programs, biases still exist [31]. Machine learning relies on training cases, and the number of cases used is often too small to avoid miscalculations [32]. Many modeling programs also rely on older, static datasets that do not use newer dynamic models [33]. Despite these known shortcomings, computational models are a workhorse in today’s protein research. Our findings highlight these potential problems.

After beginning this research and selecting the variants to test, we discovered the ARUP GALT database [34]. This database appears to be a more extensive list of GALT variants (363 missense variations) compared to ClinVar (218 missense variations) and most of the variants are listed as pathogenic. The A81T variation is perhaps the most interesting where our biochemical assay shows 52% activity while PredictSNP and EVE determine that it is benign, and yet the ARUP database lists it as pathogenic based on one of the initial publications of GALT [35]. The database does not list the H47D variation and designates the E58K variation as pathogenic based on an unpublished result.

Referencing the Galactosemia Proteins Database 2.0, we utilized the results of predictive modeling to analyze the possible effects of known missense mutations on the structure and activity of GALT [36]. Of the mutations tested in our experiments, only the A81T variant has predictive modeling results in the Galactosemia Protein Database 2.0. The results from this database indicate the A81T variant would have numerous effects on the GALT protein. These changes include altered intrachain and interchain reactions, ligand interactions, hydrogen bond interactions, and hydrophobic interactions. These changes align with the findings of our experiments which indicated significant disruption in enzymatic activity of the A81T variant of GALT.

There are a few limitations to our study. First, our enzymatic assays are only able to measure *in vitro* activity, not in vivo activity. As with any other* in vitro* study, our experiment cannot determine the true impact of these findings in vivo. Second, we tested only a small number of the more than 300 known mutations of GALT (https://www.ncbi.nlm.nih.gov/clinvar/?gr=1&term=GALT%5Bgene%5D&redir=gene). Although the results of our assays were statistically significant, conclusions about the relationship between *in silico* and *in vitro* testing for other mutations cannot be made based on our findings. Third, our research focused on mutations within regions of GALT with very few documented pathogenic variants. The accuracy of predictive bioinformatic models in these regions may not correlate strongly with their accuracy in other regions of the protein. Lastly, these *in vitro* tests do not measure other factors that may be affected by a variation like protein stability, protein mislocalization, decreased protein-protein interactions, etc. These other factors may need to be assessed to get a full picture of the effect of each variation.

## Conclusions

Significant advancements have been made in the accuracy of bioinformatic predictive modeling over recent years. As these predictive models become increasingly utilized in biomedical research, it is important to study and quantify their predictive accuracy in relation to real-world mutations. These technologies provide quick, efficient, and cost-effective techniques to discover and investigate potentially detrimental mutations in human proteins. However, the clinical value of these predictive models has been mostly unproven. We sought to design an experiment that would allow us to quantify the accuracy of these* in silico* predictive models in relation to *in vitro* assays. To determine the validity of these predictive models in future research and clinical studies, we conducted *in vitro* assays that quantified the effects of numerous mutations on the human enzyme GALT. All three variants tested (A81T, H47D, E58K) showed decreased enzymatic activity relative to native protein. When compared to the results of numerous predictive models (Molecular dynamics RMSD comparison, PredictSNP, EVE, ConSurf, SIFT), our results indicated extensive discrepancies between the predicted impact of mutations versus their actual effects on GALT function *in vitro*. We were able to quantifiably demonstrate discrepancies between predictive models and *in vitro* studies across several mutations within the GALT protein. These results indicate possible issues with relying on predictive modeling to determine the effects of mutations of protein function. Further studies to elucidate the clinical value of these predictive models are warranted, not only for GALT but also for any other proteins of human clinical significance.
